# Oxytocin increases perceived competence and social-emotional engagement with brands

**DOI:** 10.1371/journal.pone.0260589

**Published:** 2021-11-30

**Authors:** Jorge A. Barraza, Xinbo Hu, Elizabeth T. Terris, Chuan Wang, Paul J. Zak

**Affiliations:** 1 Department of Psychology, University of Southern California, Los Angeles, CA, United States of America; 2 Center for Neuroeconomics Studies, Claremont Graduate University, Claremont, CA, United States of America; University of Rome, ITALY

## Abstract

Humans express loyalty to consumer brands much like they do in human relationships. The neuroactive chemical oxytocin is an important biological substrate of human attachment and this study tested whether consumer-brand relationships can be influenced by oxytocin administration. We present a mathematical model of brand attachment that generates empirically-testable hypotheses. The model is tested by administering synthetic oxytocin or placebo to male and female participants (N = 77) who received information about brands and had an opportunity to purchase branded products. We focused on two brand personality dimensions: warmth and competence. Oxytocin increased perceptions of brand competence but not brand warmth relative to placebo. We also found that participants were willing to pay more for branded products through its effect on brand competence. When writing about one’s favorite brands, oxytocin enhanced the use of positive emotional language as well as words related to family and friends. These findings provide preliminary evidence that consumers build relationships with brands using the biological mechanisms that evolved to form human attachments.

## Introduction

Humans are unusual in that they are gregariously social. Social animals have evolved neural mechanisms that process a rich set of information from interacting conspecifics. One of the more recent findings that helps explain human sociality is the role of the neuromodulator oxytocin [1,2]. Oxytocin (OT) facilitates attachment to other humans [3], animals [4,5] and perhaps objects [6]. People will often describe their favorite objects using attachment-related language like "love" or "need" [7,8]. People’s descriptions of brands such as Apple, Amazon, and the Walt Disney Company use similar emotional language and may even have perceived "personalities” [9]. Consumers prefer brands described with emotional language [10–13]. Yet this apparent anthropomorphism could simply be an artifact of the paucity of linguistic terms to describe feelings towards objects.

Companies create marketing that seeks to endow brands with positive attributes including trust, competence, and warmth in the belief that this builds customer loyalty [14–17]. Self-reported data is known to be inaccurate [18], but until recently it was seen as the best way to explore brand relationships. An alternative is to assess brand attachment, if it exists, using techniques from neuroscience. While most of the research in consumer neuroscience has focused on preferences and choices [19,20], a study measuring peripheral and central nervous system responses showed attachment for favorite brands produced patterns of activity similar to responses seen for loved ones [21]. The findings for greater arousal in the palms (electrodermal activity) and increased BOLD (blood-oxygen-level dependent) signal in the insula for preferred versus neutral brands is not definitive because such neurophysiologic responses occur for a wide range of stimuli rather than being narrowly focused on attachment [22,23].

The biology of attachment has been informed by techniques to measure the brain’s release of, and to pharmacologically manipulate, the neurochemical oxytocin (OT) in human. The brain releases OT after positive social interactions that undergird attachment and facilitates trustworthiness [24,25], donations to charity [26], and eliminates out-group biases [27]. Administration of exogenous OT increases trust in strangers [28], generosity [29], charity [30], improves the ability to understand others [31,32], and may treat psychiatric disorders that produce social pathologies [1,33].

There are some concerns regarding the robustness of findings in intranasal OT studies. The effect of OT administration on trust failed a meta-analysis due in part to low statistical power [34], heterogeneous effects on participants [35], and lack of robustness across trust tasks [36]. Similarly, the effect of exogenous OT on mind reading could not be replicated [37]. A large sample (N = 254) registered replication of Kosfeld et al. (2005) [28] identified that OT affects trust when eliminating methodological confounds but only for a subset of participants who show low trustworthiness [38].

Nevertheless, there is substantial evidence that intranasal OT crosses the blood–brain barrier in humans and nonhuman primates using a variety of analytical techniques [39–41]. Moreover, intranasal OT appears to accumulate in brain regions that show OT mRNA expression (striatum, amygdala, thalamus, hypothalamus) [42] and that impact social behaviors [43]. These findings are complemented by studies that measure endogenous release of OT during a social task and then demonstrates that exogenous OT administration influences behavior in the same or similar task. This has been done for interpersonal trust [25,28,43] and charitable giving [30,44]. More generally, the prosocial effects of intranasal OT have been shown for a variety of situations, including punishment of free-riders [45] and the representation of social value [46] providing confidence that OT influences prosocial behaviors. We hypothesized, based on the conservative nature of evolution [47], that the biological basis for human-to-human relationships may also facilitate relationships that people appear to have with brands.

Fürst et al. (2015) [48] published the first study of OT in brand attachment. The authors reported that OT administration increased ratings of favorite brands but only for healthy participants below the median value for autism-spectrum quotient (AQ) scores and OT *reduced* self-reported attachment for participants with above median AQ scores of 14. Slicing by median AQ calls into question the robustness of the findings as does depending completely on self-report for OT’s impact rather than use an objectively observable behavioral assay to measure attachment. Both Fürst et al. [48] and the current research examine the consumer-brand relationship. However, relationships are multi-faceted so the present study examined several dimensions of brand relationships in order to generate convergent evidence for the effect of OT on brand attachment. These dimensions include perceptions of warmth and competence, the use of social-emotional language when describing brands, and the amount people are willing to pay for branded products.

### A model of brand attachment

In order to understand how brand attachment affects purchasing decisions and to generate specific testable hypotheses, we propose the following standard two-good choice model from economics [49] with the inclusion of an attachment parameter. Let α > 0 denote the factors that impact brand attachment. The model guided the experimental design and analysis by clarifying the pathways through which attachment affects consumption when consumers can choose between similar goods to which they are attached and nonattached.

Consumers can choose between good c_1_ for which they have formed an attachment or a competing good of equal quality c_2_ for which no attachment has been formed. Consumers seeking to obtain the services of these goods solve the following utility optimization problem,

$${\text{Max}}_{\text{c}1,\;\text{c}2}\;\text{U}({\text{c}}_{1},\;{\text{c}}_{2};\;\alpha )$$

$$\text{s}.\text{t}.\;{\text{p}}_{1}{\text{c}}_{1}+{\text{p}}_{2}{\text{c}}_{2}=\text{M},$$

where p_1_ and p_2_ are the prices of each good, assuming p_1_>p_2_>0, M>0 is the consumer’s budget, and U(c_1_, c_2_) is a standard increasing, continuous, and concave utility function. The implications of this model can be seen concretely by using the following utility function,

$$\text{U}({\text{c}}_{1},\;{\text{c}}_{2};\;\alpha )\;=\alpha \;\text{ln}\left({\text{c}}_{1}\right)+\text{ln}\left({\text{c}}_{2}\right).$$

The optimal consumption of each good can be found by substituting the constraints into the objective function and differentiating. As shown in the Appendix, the desired consumption of good c_1_, call it c_1_*, is

$${\text{c}}_{1}*=\frac{\alpha M}{\left(1+\alpha \right)\text{p}1}.$$

The Appendix shows that consumption of c_1_* increases with brand attachment (α), that more of c_1_ is purchased when the consumer’s budget increases (M), and the demand for c_1_ declines as its price (p_1_) increases. The Appendix also proves that people are willing pay more for the good (p_1_) to which they have an attachment as brand attachment increases (α). While our data were collected before pre-registration of studies became common [50], the model constrains the analysis to be hypothesis-driven rather than post-hoc.

We operationalize these implications by identifying how to measure brand attachment and the purchase price one is willing to pay for a branded product.

#### Trust, warmth, and competence

Attachment among human beings depends on social-emotional processes including trust, reliability, and warmth [51,52]. OT may influence these aspects of relationships [28,53]. Trust also builds brand relationships by promoting commitment, satisfaction, and loyalty [14,54]. Research in marketing has shown that people appear to anthropomorphize brands, assigning to them warmth, intentions, and competence, perceptions that map closely to two factors of trust: benevolence (concern/care) and competence (ability, expertise, knowledge) [55,56]. At the same time, judgments of warmth and competence affect perceptions of a brand [8,57,58]. Thus, one way to assess brand attachment is to evaluate the impact of OT on perceptions of a brand’s warmth and competence.

#### Social-emotional language

Analyzing language is another measure of the relationship to a brand. People use more words when writing about a topic in which they are interested compared to topics of less interest [59] and words associated with positive emotions indicate relationship stability [60]. Negative emotional language, on the other hand, indicates relationships of low quality [61,62]. OT affects social salience [53] and social language [63] and may therefore impact how, and how much, people write when describing a brand to which they are attached.

#### Willingness to pay

A standard approach in economics and marketing to infer one’s preferences is known as willingness-to-pay (WTP). WTP is the maximum price one at which one is willing to purchase a product. While there are several ways to assess WTP, the approach that provides the most accurate predictions of purchasing behavior uses a lottery over products [64]. In this approach, a participant first states his or her WTP and then a public lottery determines the selling price of the product. If the random price is less than or equal to the person’s WTP he or she must purchase the good [65]. This approach will allow us to test the model’s prediction that attachment to a brand increases WTP.

#### Neurologic intervention

Mammalian social attachment depends on the brain’s production of OT and the neural pathways it activates [1,66]. We manipulated OT pharmacologically to test if OT affected attachment to brands and the purchase price one is willing to pay for branded goods.

Exogenous OT administration appears to produce more accurate recognition for familiar compared to unfamiliar faces [67]. As such, we expected that intranasal OT would produce stronger attachment (α) to brands to which participants had been previous exposed. We also hypothesized that OT would increase people’s perceptions of brand warmth, competence, and willingness-to-pay for brands after exposure similar to a brand advertisement, compared to participants receiving a placebo. A social cue or contact is generally necessary for OT to affect behavior [1,68], as OT may facilitate the encoding process of social cues [31,53]. Brand-focused advertisements were used because they are a common vehicle for brand communication and can be easily controlled in an experimental setting. We also expected that OT would influence the language used to describe brands, including the amount written, how the brand was discussed in the context of social relationships, as well as the amount of positive affect used in writing.

## Method

Seventy-seven females (51.3%) and males (48.7%) from liberal arts colleges and the surrounding community (Southern California, US) participated in this study. Participants (ages 18–53, M = 23.56, SD = 7.32) were randomly assigned to receive either synthetic OT (Monarch Pharmaceuticals, Bristol, TN; n = 39) or placebo (n = 38) in a double-blind design. All participants were screened by a medical professional for possible contraindications. Exclusion criteria included any history of mental illness, cardiac disorders, kidney dysfunction, and pregnancy or possible pregnancy. Females were only included after a negative urine pregnancy test. Note that our sample size is moderate in order to limit possible adverse effects from drug administration. This is offset by collecting multiple observations per participants. Reported ethnicities for the study sample were 44% white, 22% Asian, 12% Hispanic/Latino, 10% Black, 12% Other. The Institutional Review Board of Claremont Graduate University approved this study (#2429) and all participants gave written informed consent prior to inclusion. All research was performed in accordance with relevant regulations and the guidelines in the Declaration of Helsinki. Study participation was approximately 90 minutes with $30 USD compensation. All survey and tasks were computer mediated (e.g., keyboard, mouse responses). There was no deception of any type

### Baseline brand assessment

In order to establish perceptions of warmth and competence [69], brand personality (Brand Personality scale), and familiarity (e.g., “How well do you know the brand Lexar?”), participants completed an online survey at least 24 hours prior to participation, with most completing 1-week prior (*M* = 8.3 days, SD = 10.03). Assessments were reported on Likert-type scales anchored by 1 (not at all) and 7 (very much). Twelves brands were included from three product categories: USB flash drives (Kingston, PNY, Lexar, Sandisk), reusable water bottles (Contigo, Nalgene, Sigg, Kleen Kanteen), and portable headphones (Sennheiser, Klipsch, Skull Candy, AKG). These categories were selected as they have wide appeal, include brands that are not universally well known, and can be purchased with study compensation. These three criteria reduce the likelihood that existing brand attitudes would impact results and potential product purchases could be made.

### Oxytocin administration and survey

Participants visited the lab for the main portion of the study. After consent and medical clearance, participants were seated at partitioned computer stations and completed surveys that measured demographics (e.g., age, gender, ethnicity), personality (Interpersonal Reactivity Index, IRI; Five-Factor Inventory) [70,71], and emotional state (Positive and Negative Affect Schedule, PANAS) [72]. The surveys are included to control for possible trait differences. Participants were next administered either OT (40 IU) or placebo (normal saline similar in odor and taste) intranasally by a naive experimenter using prior double-blind protocols [29]. Our lab obtained investigational new drug (IND) approval from the U.S. Federal Drug and had our pharmacy load OT (Monarch Pharmaceuticals, Bristol, TN) into standard aerosolizers as in our previous studies. Participants then completed a 40-minute filler task (e.g., providing demographic information and other survey content) to allow OT to reach the brain [40,41].

### Brand exposure and assessment

Participants were exposed to statements about brands taken from company websites with brand logos for one-half of the brands (6 of the 12 brands randomly selected, 2 brands per product category; see Appendix for examples). Each statement was developed from the company’s mission and history and edited to contain similar content (e.g., topics, language, valence), and length (79–81 words). Statements were presented in Qualtrics software for 30 seconds before automatically proceeding to the next brand statement. After the brand exposure task, all 12 brands (half exposed brands, half unexposed brands) were evaluated for brand personality, warmth, and competence as in the baseline brand assessment.

### Willingness-to-pay

Branded products’ values were assessed by asking participants to estimate the retail price and the price they would pay to purchase each product. Decisions were authentic because participants were asked to use their $30 in compensation to bid on each product using a modified Becker–DeGroot–Marschak method (BDM) [64]. The price of every product was determined randomly and publicly after bids were placed by a draw from a uniform distribution between 1–30 by pulling cards with replacement from an urn. For transparency, every participant whose bid equaled or exceeded the random price was required to purchase the product at the random price; all others were not allowed to buy the product [73]. The lottery was held at the conclusion of each experimental session after all data were collected. Bids were neither required nor encouraged. All products were available for purchase and were delivered to participants at the end of the experiment.

### Writing task

Participants were asked to write a short story about an experience they had with their favorite or preferred brand, why they chose that brand, and why it was special to them [9]. No time or character limits were placed on responses. Responses were analyzed using the Linguistic Inquiry and Word Count (LIWC) program (60). LIWC measures the frequency of specific word types. We measured the overall word count, social language (family, friends), and emotional language. In the social category, we quantified subcategories for friends (e.g. buddy, neighbor) and family (e.g., daughter, husband), while in the emotion category we assessed subcategories for positive affect (e.g., love, nice, sweet) and negative affect (e.g., hurt, ugly, nasty). The LIWC has been shown to be a statistically valid method to capture emotional responses [74].

### Dismissal

After completing all tasks, participants were told the results of the BDM bids, given any items they purchased, and paid their remaining earnings in private. Participants had the opportunity to discuss the experiment and its goals with the experimenters if they so chose.

### Analysis strategy

Composite measures for brand categories and products were created because we did not have hypotheses for individual items. Analyses by product category (water bottle, USB drives, earphones) produced identical results to those for the aggregated dependent variable (S1 File). The effects of OT were analyzed using ANOVA and t-tests were used to assess differences in means. Several dependent variables were analyzed to test for the robustness of findings. The power calculation was based on the hypothesis that OT would increase willingness to pay for branded items to which they had been exposed and the effect size (48%) from an intranasal OT study on donations to charity ([30]). The total number of observations for brand effects was 444 (37*12) that produced a power of test of 0.99 using G*power [75].

### Data availability

The data and codebook are owned by the authors and can be freely downloaded at Open ICPSR-153381, DOI: https://doi.org/10.3886/E153381V2.

## Results

Those in the OT and placebo groups were not significantly different in measures of personality and mood (Big Five, IRI, PANAS; *ps* >.10). Random assignment led to more males than females in the OT condition compared to the placebo condition (t = 2.20, p = .03) so all analyses were checked for differences caused by sex. Participants rated most brands as low in familiarity (*M* = 2.83). None the analyses below significantly change when controlling for sex or brand familiarity.

### Brand competence, warmth, and personality

**Fig 1:**
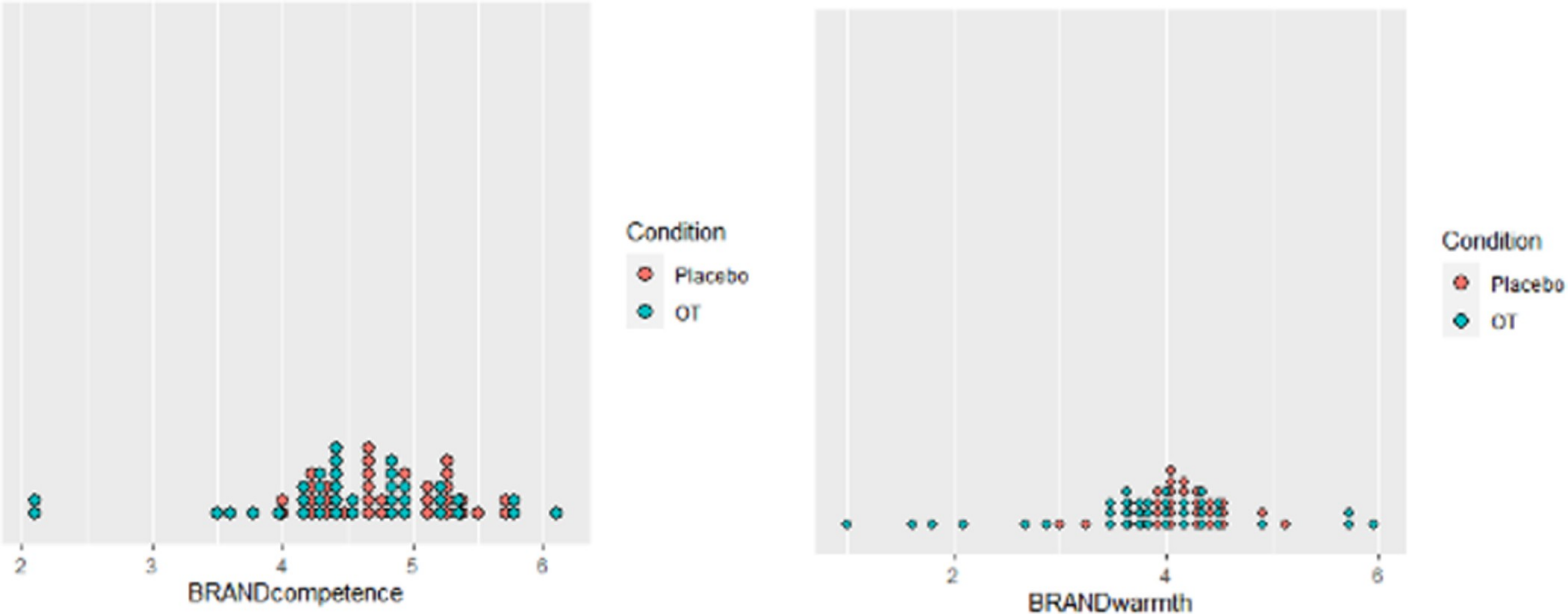
The distribution of brand competence and warmth by condition. The change in competence after brand exposure was 132% higher for OT compared to placebo (17.6% vs. 7.6%; p = .05).

A 2 (treatment: OT/placebo) by 2 (time: baseline/post exposure) repeated-measures analysis of variance (ANOVA) analysis was used in order to examine differences in the brand perceptions of OT and placebo groups after brand exposure. Across exposed brands, both OT and placebo conditions increased in brand warmth from pre to post exposure (*F* = 26.71, *p* < .001, η_p_^2^ = .28). No significant differences were found between OT and placebo as expected and there was no interaction effect by condition (*F* = .34, *p* = .56). Exposure to brands also increased brand competence from pre to post exposure across both conditions (*F* = 35.01, *p* < .001, η_p_^2^ = .38). As predicted, there was a significant interaction by condition (*F* = 4.08, *p* = .04, η_p_^2^ = .06). OT increased competence for exposed brands compared to placebo (*OT M* = 17.6%, Placebo *M* = 7.6%; *t* = 1.99, *p*< .05, *d* = .47, 2-tailed). Gender did not impact the results (Welch’s t-test, t = 1.116, p = .13). Fig 1 illustrates this finding.

To test whether OT also affected other brand personality dimensions, we compared OT and placebo conditions pre- and post-exposure to measures of excitement, sophistication, and ruggedness (sincerity and competence from Aaker (1997) [76] were removed given the overlap with warmth and competence). There were no significant main or interaction effects across these brand personality measures (*ps*>.05).

We tested non-exposed brands to see if exposure was necessary for OT to influence brand perceptions. There was no effect of OT on brand warmth (*F* = .06, *p* = .80), brand competence (*F* = .01, *p* = .90), or other brand personality measures (*ps*>.05).

### Willingness-to-pay and price estimates

As predicted by the mathematical model, WTP was affected by brand attachment. The data reveal a significant and positive correlation between brand competence and WTP (r = .13, *p* = 0.007). Separating exposed and non-exposed brands showed that the relationship between competence and WTP is due, as expected, from exposure to brands (Exposed r(WTP, competence) = .11, p = .02; Non-Exposed r(WTP, competence) = .03, p = .52). No interaction effect was found between the product of OT and exposed brand competence on WTP (p>.05). Consistent with the mathematical model, OT does not directly affect willingness-to-pay across the two conditions (OT M = $3.61, Placebo M = $4.79; *F* = 0.33, *p* = 0.56) nor for exposed brands (F = 0.02, p = 0.88) in an ANOVA. The gender of participants receiving OT did not impact WTP (p = .15). In addition, OT did not affect the estimated retail prices of products (OT = $2.19, Placebo = $2.09, F = 0.39, p = 0.68) Further, OT did not affect estimates of retail prices for any product category or for exposed versus unexposed brands (S1 File).

### Writing task/brand engagement

**Fig 2:**
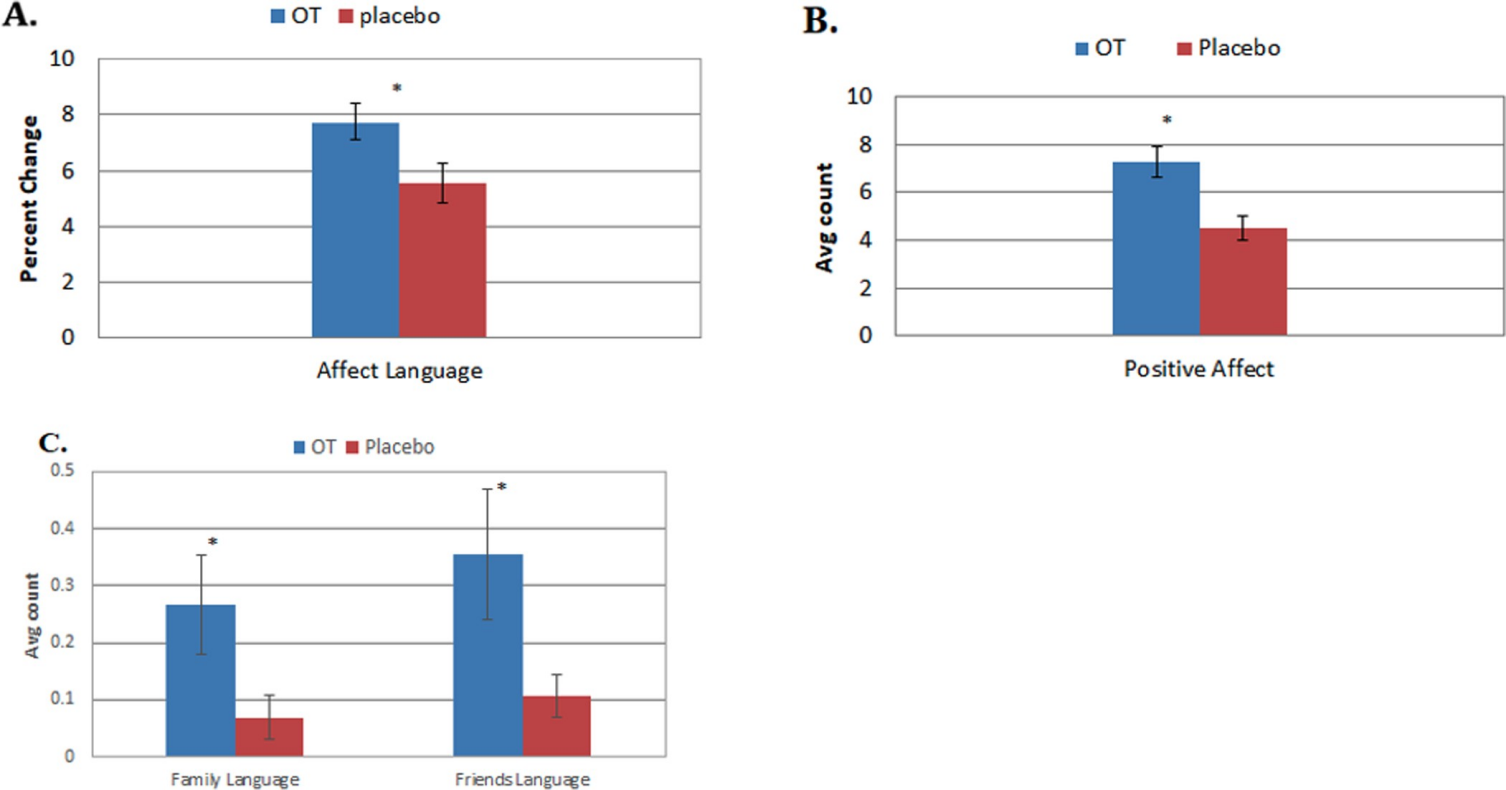
The effects of OT when writing about brands with standard errors. A. OT increased the use of affective language by 39% (p = .021). B. This was driven by a 62% increase in positive affective language (p = .001). C. OT also boosted discussions of family about 286% (p = .04) and friends by 227% (p = .04) in participants’ writing.

In order to confirm the main hypotheses, participants were asked to write about a favorite brand. Affective language use when writing about a favorite brand was higher for OT relative to placebo (OT M = 7.72, placebo M = 5.56, t = 2.35, p = .021, *d* = .56; Fig 2A) supporting our hypothesis. Analyzing valence, we found that OT significantly increased the use of positive affective language (OT M = 7.27, placebo M = 4.49, t = 3.50, p = .001, *d* = .83), but not negative affective language (OT M = .37, placebo M = .53, t = .858, p = .39; Fig 2B). This effect was largely generated by women (p = .001). There was no effect of OT on the length of brand essays (OT M = 30.91, placebo M = 34.22, t = .689, p = .49), or the frequency of overall social language (OT M = 6.93, placebo M = 5.63, t = 1.34, p = .18). But, OT amplified how often participants mentioned family (OT M = 0.27, placebo M = 0.07, t = 2.07, p = .04, *d* = .49) and friends (OT M = 0.36, placebo M = 0.11; t = 2.06, p = .04, *d* = .49; Fig 2C) compared to placebo.

An inter-rater reliability analysis was conducted in order to examine the accuracy of LIWC in correctly classifying words. Three independent raters were trained to classify words across LIWC categories (e.g., positive and negative affect, relational words). Unlike LIWC, raters were instructed to take the context of the word into account (e.g., use of sarcasm). There was high agreement between raters, with the lowest average measure at .776 for the family category (all others .826-.949) with a 95% confidence interval .285 to .905 (F(76,152) = 10.55, p < .001). Aggregating raters and comparing them to LIWC, there was high agreement; the lowest average measure was .737 for friends with a 95% confidence interval from .586 to .833 (F(76,76) = 3.78, p < .001). All others had average measures between .756 - .872 (ps < .001). The human rater data corroborates the LIWC findings.

## Discussion

Our findings demonstrate that OT influences brand attachment by increasing perceived competence and the use of social and emotional language when describing brands. There is some evidence that OT increased WTP for branded products indirectly through its impact on competence as predicted by the mathematical model we developed. Our analysis showed that a 10% increase in competence increased the bids for branded products by 2.5%. Put another way, a one standard deviation increase in perceived competence (1.01) caused people to bid $2.30 more for the good on offer. This result was found via correlations but was not confirmed in a mediation test. The increase in positive affective language when describing brands for participants on OT provides corroborating support for the effect of OT on brand attachment.

Participants appeared to be cognitively intact because there was no effect of OT on estimates of retail prices for products. Instead, OT appears to have increased attachment to brands, at least temporarily and in this way affected WTP for products. The impact of OT went beyond the mere exposure effect in which both warmth and competence increased after participants received information about brands. While OT did not affect perceived warmth using a standard scale, OT did affect the quantity of positive emotional language used to describe preferred brands and the use of language reflecting close personal relationships. The bulk of brand exposure conveyed warmth (see Appendix), for example, "people who care about the quality of products…," and "…respect, loyalty, and integrity are a vital part of our success" that could induce the endogenous release of OT suppressing the effect of exogenous OT.

The data showed that OT increased perceptions of brand competence beyond the exposure effect. Brand exposure was positively framed, and that could have led to positive, rather than negative, brand perceptions after OT administration. Intranasal OT’s effect on social cognition is dependent on a social cue [68] and contextual cues [77,78]. Indeed, intranasal OT appears to affect beliefs based on the framing given to participants [38,45,68]. While one might expect OT to affect warmth more than competence, positive exposure cues may have affected both these cognitive perceptions [79]. The impact of OT on competence but not warmth may be due to ceiling effects as brands had higher warmth ratings than competence ratings. From a practical perspective, brand warmth may be less important to marketers than brand competence as the latter influences brand admiration and purchases [58,69].

Given the inconsistent effects of OT on social behaviors in the literature, the results should be considered preliminary until replicated [80]. While there is concern about the state of intranasal OT research that is leading some to lose “trust” in published findings [35], expanding the set of behaviors that intranasal OT influences, as we have done here, adds value to this evolving literature. In a similar vein, research measuring endogenous OT was affected by calls to dismiss findings from unextracted assays (e.g., [81–83]) that is being re-evaluated with a better understanding of OT measurement [84], extraction methods, and how to associate the change in OT with social behaviors (e.g., [85,86]. Extension and replication are essential for both intranasal OT studies and endogenous OT measurement studies.

There are several practical applications of this research. Companies could use our findings to build brand attachments via both competence and warmth/positive affect, though only the former motivates people to pay higher prices for branded products consistent with prior research [58,73,87]. When customers have personal emotional connections to brands, companies are often, though not always, able to sustain sales and pricing power [15]. As a result, the investments companies make in building brand personalities, especially those that focus on competence and positive affect are likely to have a positive return. We caution, though, that our experimental design only tested one product per brand. Future research should identify how portfolios of products influence perceived competence and warmth of brands as well as pricing.

Our findings for how people wrote about preferred brands could be used by companies to build brand trust and personalities. The increased use of positive affective language as well as mentions of family and friends due to OT might provide a way for consumers to promote brand attachments using social media. The ubiquity of social media combined with the power of personal recommendations suggest that companies should facilitate customers sharing thoughts about their favorite brands by including "share" links in all communications. This effect could be accentuated by holding brand-building events where consumers who "love" a brand can interact with each other and potentially influence less-attached consumers.

The results of this research also extend reports that intranasal OT increases attributions of relationship qualities to brands [48]. Future research can extend our findings by exploring the extent to which OT impacts other aspects of consumer behavior. For example, a study of online shopping found that plasma OT increased by 14% after the receipt of a coupon while shopping while OT did not change for participants who did not receive a coupon [88]. These findings indicate that the shopping experience itself can build attachment to brands. Indeed, other work has found that OT increases attention to socially relevant information [53] suggesting that shopping or advertising activities that induce the brain to release OT may strengthen brand attachments and loyalty [89].

This study’s demonstration that consumer-brand “relationships” may be more than just a metaphor provides insights into human social behaviors. The conserved human social attachment system appears to be part of the neurologic process through which consumers build relationships to brands. OT administration increases attributions of human qualities to non-living objects, consistent with our findings. We showed that OT enhanced positive affect when writing about a brand, something that occurs even without OT administration [90,91]. Brand attachment creates loyalty and price insensitivity that are valuable to companies. Consumers who have relationships to brands also benefit through increased satisfaction when using favorite branded products and reduced cognitive load relative to choosing among the plethora of available consumer goods.

## Supporting information

S1 File (DOCX)
