# Supplementary material for: Oxytocin increases perceived competence and social-emotional engagement with brands
Source: PLoS One. 2021 Nov 30;16(11):e0260589. doi: 10.1371/journal.pone.0260589 (PMC8631632; doi:10.1371/journal.pone.0260589)
Supplement: S1 File — (DOCX) [file pone.0260589.s001.docx]

Brand exposure examples

**WATER BOTTLES**

**Klean Kanteen**

Our story started in 2004 when we provided people with an affordable alternative to plastic bottles with the world’s first BPA-free metal hydration bottle. Klean Kanteen is a small family owned company with an enduring commitment to our products, customers, and the environment. Our mission is to provide affordable, safe, healthy, high quality products and to promote and encourage healthy, sustainability, and environmental awareness. Today Klean Kanteen continues to lead through innovation and environmental education.

**Contigo**

Why Contigo? Our technology is based on your needs and our own personal experiences. We come up with a cool new technology to support the innovation and then we test it over and over again to make certain it's of superior quality. We are forward-thinking inventors who value creative, unconventional ideas. We are fully dedicated to what we make, how we make it, for whom we make it, and the way it impacts people’s lives and the environment.

**Nalgene**

In the 1970s, Marsh Hyman heard his Nalgene storage bottles were being used by Boy Scouts for hiking in the woods. From there, Nalgene was born. Today, we are helping people simplify their lives while respecting the environment, creating reusable containers of all kinds, made from materials to suit your personal preferences, needs and lifestyle. So if you’re looking for a virtually indestructible drinking bottle for a backcountry adventure you’ll find a Nalgene product that gets the job done.

**Sigg**

For over 100 years, we have utilized Swiss precision to design and build products that make life easier and a little more enjoyable. We are a brand of optimism, of forward thinking, and of people that care about the quality of the products they build. We make bottles to the highest standards of quality; bottles that are ecologically sound, reusable and easily recycled, as well as fashionable. We see a future that is green and clean, sleek and stylish.

**USB FLASH DRIVES**

**Kingston**

In 1987, we entered the market with a single product that redefined industry standards for years to come. Reliability is the cornerstone of our USB flash drives. We combine the most extensive and stringent testing processes in the memory industry with an exceptional free tech support center. We have continually set industry standards of quality and reliability throughout our history. Kingston’s tenets of respect, loyalty, flexibility, and integrity are a vital part of our success.

**Lexar**

For more than 15 years, we have been trusted as a leading global producer of memory drives. Our memory solutions provide the performance and reliability needed to get the most from your digital device. We know that photographs, videos, and documents are more than just files to you. Our products undergo extensive testing in our Lexar Quality Labs to ensure performance, quality, compatibility and reliability. We are committed to providing outstanding customer service, with personalized advice and expert support.

**PNY**

PNY has 30 years of experience serving consumers who care about quality and responsible business practices. Our products function with a wide variety of computer and digital devices to provide a wide range of end-user solutions. Our array of storage solutions provides outstanding speed, performance, and reliability. We use eco-friendly product packing made from environmentally sustainable materials and oriented using soy-based ink. We provide customers with quality products that are reliable, affordable, and geared towards consumers’ needs.

**SanDisk**

- SanDisk drives the future of storage solutions. Every day, millions of people count on the performance, quality, and reliability of SanDisk products. Our leading-edge flash drives enable fast, reliable access to data. From important documents to irreplaceable photos, much of what we value is stored digitally. Our rigorous quality control and first-class engineering teams deliver products you can trust with your precious data. It's all part of delivering on our mission to enrich people's lives through digital storage anytime, anywhere.

**IN-EAR HEADPHONES**

**Klipsch**

Our story started in 1946 when Paul W. Klipsch, genius and maverick, designed and hand-built speakers with the goal of bringing live music into his living room. Today, our products have become the stuff of legends. Our world-class speakers and headphones allow people to connect with their favorite music like no other brand by delivering the power, detail, and emotion of the live music experience. We are knights and humble servants of audio. We are Keepers of the Sound.

**Sennheiser**

We are the scientists and artists of sound shaping the future of the audio industry, based on our history, our culture of innovation and our passion for excellence. For us, good sound is not enough. Sounds can transport us to distant places, awaken sleeping emotions and even intensify our sense of taste. We provide audiophiles with the most comfortable studio-quality audio on the planet. We are family-owned, sustainable, and unmatched in our commitment to music lovers everywhere.

**Skull Candy**

Skull Candy is a leading audio brand that reflects the collision of the music, fashion and action sports. Our logo symbolizes our motto, “Every revolution needs a soundtrack.” Founded on innovation, we fuse bold color schemes, loud patterns, unique materials and creative packaging with the latest audio technologies and innovative functionalities. We work to open up a world of sound where you play, at the best snow peaks, skate parks and surf breaks across the world.

**AKG**In the world of pro audio, AKG is synonymous with excellence. Our passion for sound makes us a leading maker of high-quality headphones. We work hard to deliver the powerful, undistorted sound that music professionals have relied on for more than 60 years. Our commitment to our customers motivates us to consistently produce the truest and most natural-sound in the history of audio. We have been impacting lives with vibrant, true audio for the better part of a century.

**Data is separated by product category (water bottle, USB drives, headphones).**

| Warmth | USB | | Water bottle | | Headphones | | Aggregated | |
| --- | --- | --- | --- | --- | --- | --- | --- | --- |
|  | Mean | SD | Mean | SD | Mean | SD | Mean | SD |
| Placebo | 3.992 | 0.955 | 4.369 | 1.054 | 4.072 | 0.996 | 4.147 | 1.010 |
| OT | 3.796 | 1.286 | 4.028 | 1.425 | 3.579 | 1.407 | 3.804 | 1.383 |
| t-test | 1.423 |  | 5.612 |  | 5.158 |  | 4.049 |  |
| p-value | 0.076 |  | 0.011 |  | 0.000 |  | 0.000 |  |
| Competence | USB | | Water bottle | | Headphones | | Aggregated | |
|  | Mean | SD | Mean | SD | Mean | SD | Mean | SD |
| Placebo | 4.840 | 1.088 | 4.985 | 1.047 | 4.788 | 1.077 | 4.873 | 1.072 |
| OT | 4.622 | 1.269 | 4.517 | 1.422 | 4.411 | 1.307 | 4.517 | 1.362 |
| t-test | 1.501 |  | 3.118 |  | 2.492 |  | 4.142 |  |
| p value | 0.066 |  | 0.001 |  | 0.006 |  | 0.000 |  |
| WTP | USB | | Water bottle | | Headphones | | Aggregated | |
|  | Mean | SD | Mean | SD | Mean | SD | Mean | SD |
| Placebo | 19.405 | 12.898 | 21.857 | 15.244 | 38.317 | 29.842 | 26.526 | 22.310 |
| OT | 20.467 | 27.331 | 21.633 | 13.443 | 34.731 | 30.423 | 25.641 | 38.802 |
| t-test | -0.429 |  | 0.135 |  | 1.030 |  | 0.571 |  |
| p value | 0.666 |  | 0.446 |  | 0.152 |  | 0.284 |  |

**Appendix: Solution to the Mathematical Model**

A logarithmic function is strictly increasing and concave and thus the contraints can be directly substitutted into the maximand to find the maximum. Thus, one differentiates

Max_c2_ α ln($\frac{M- p2 c2}{p1}$) + ln(c_2_).

The first order condition is necessary and sufficient for an optimum, producing

c_1_* = $\frac{\alpha M}{(1+\alpha)p1}$.

By inspection, we can see that consumption of the good with the brand attachment, c_1_*, is increasing in the budget M and decreasing in its own price, p_1_. The impact of brand attachment on c_1_* can be found by differentiation,

$\frac{dc1*}{d\alpha}=\frac{M}{(1+\alpha)p1}$ > 0.

This shows that the consumption of c_1_* is increasing with brand attachment α.

To prove that price increases with brand attachment α, one can take the total differential of c_1_* with respect to α and p_1_. Setting the total differential equal to zero since c_1_* is an optimum produces

$\frac{dp1}{d\alpha}=\frac{p1}{\alpha(1+\alpha)}$ > 0.
